# Impact of gut microbiome on serum IgG4 levels in the general population: Shika-machi super preventive health examination results

**DOI:** 10.3389/fcimb.2023.1272398

**Published:** 2023-10-16

**Authors:** Aoi Koshida, Shigehiro Karashima, Kohei Ogura, Yuna Miyajima, Kazuhiro Ogai, Ren Mizoguchi, Yasuo Ikagawa, Satoshi Hara, Ichiro Mizushima, Hiroshi Fujii, Mitsuhiro Kawano, Hiromasa Tsujiguchi, Akinori Hara, Hiroyuki Nakamura, Shigefumi Okamoto

**Affiliations:** ^1^ Institute for Frontier Science Initiative, Kanazawa University, Kanazawa, Japan; ^2^ Institute of Liberal Arts and Science, Kanazawa University, Kanazawa, Japan; ^3^ Department of Health Promotion and Medicine of the Future, Kanazawa University, Kanazawa, Japan; ^4^ Department of Clinical Laboratory Science, Faculty of Health Sciences, Institute of Medical, Pharmaceutical and Health Sciences, Kanazawa University, Kanazawa, Japan; ^5^ Department of Bio-engineering Nursing, Graduate School of Nursing, Ishikawa Prefectural Nursing University, Kahoku, Japan; ^6^ Department of Rheumatology, Kanazawa University Hospital, Kanazawa, Japan; ^7^ Department of Hygiene and Public Health, Graduate School of Advanced Preventive Medical Sciences, Kanazawa University, Kanazawa, Japan

**Keywords:** *Megasphaera*, immunoglobulin G4, causal relationship, direct linear non-Gaussian acyclic model, gut microbiota, IgG4-related disease

## Abstract

**Introduction:**

Immunoglobulin G4 (IgG4) is a member of the human immunoglobulin G (IgG) subclass, a protein involved in immunity to pathogens and the body’s resistance system. IgG4-related diseases (IgG4-RD) are intractable diseases in which IgG4 levels in the blood are elevated, causing inflammation in organs such as the liver, pancreas, and salivary glands. IgG4-RD are known to be more prevalent in males than in females, but the etiology remains to be elucidated. This study was conducted to investigate the relationship between gut microbiota (GM) and serum IgG4 levels in the general population.

**Methods:**

In this study, the relationship between IgG4 levels and GM evaluated in male and female groups of the general population using causal inference. The study included 191 men and 207 women aged 40 years or older from Shika-machi, Ishikawa. GM DNA was analyzed for the 16S rRNA gene sequence using next-generation sequencing. Participants were bifurcated into high and low IgG4 groups, depending on median serum IgG4 levels.

**Results:**

ANCOVA, Tukey’s HSD, linear discriminant analysis effect size, least absolute shrinkage and selection operator logistic regression model, and correlation analysis revealed that *Anaerostipes*, *Lachnospiraceae*, *Megasphaera*, and *[Eubacterium] hallii* group were associated with IgG4 levels in women, while *Megasphaera*, *[Eubacterium] hallii* group, *Faecalibacterium*, *Ruminococcus.*1, and *Romboutsia* were associated with IgG4 levels in men. Linear non-Gaussian acyclic model indicated three genera, *Megasphaera*, *[Eubacterium] hallii* group, and *Anaerostipes*, and showed a presumed causal association with IgG4 levels in women.

**Discussion:**

This differential impact of the GM on IgG4 levels based on sex is a novel and intriguing finding.

## 1 Introduction

Immunoglobulin G4 (IgG4) is a protein involved in immunity and the body’s resistance system against pathogens, such as bacteria and viruses (Davies and Sutton, 2015; Maslinska et al., 2022). Although IgG4 is the least common human Immunoglobulin G (IgG) subclass in the serum, IgG4-related diseases (IgG4-RD) are intractable diseases that result in elevated levels of IgG4 in the blood. These diseases cause swelling and inflammation in various tissues throughout the body, including organs like the liver, pancreas, kidneys, blood vessels, tear glands, and salivary glands (Wallace et al., 2020; Umehara et al., 2021). The development of IgG4-RD is characterized by the infiltration of lymphocytes, IgG4-positive plasma cells, and fibrosis, which leads to simultaneous or sequential swelling, nodules, and thickened lesions in multiple organs (Lu et al., 2021). The regulation of IgG4 production generally involves CD4 follicular T helper cells, T regulatory cells, and Th2 cells, with interleukin-4 (IL-4) and IL-13 promoting IgG4 and Immunoglobulin E (IgE) production (Lanzillotta et al., 2020). However, the underlying cause of elevated IgG4 levels remains unclear.

In recent years, there has been increasing attention in medical research towards the gut microbiota (GM). It has been found that the GM plays a crucial role in maintaining human health and influencing the development of diseases (Clemente et al., 2012; Yatsunenko et al., 2012; Chen et al., 2021). Additionally, the GM is involved in maintaining the delicate balance between host defense and immune tolerance and is believed to have a substantial impact on the pathogenesis of autoimmune diseases and allergies (Yatsunenko et al., 2012; Jiao et al., 2020; Xu et al., 2022). For instance, a human intervention study by Wastyk et al. showed that consuming highly fermented foods increased the diversity of the GM and reduced levels of inflammatory markers, such as IL-6 and IL-10 (Wastyk et al., 2021). Furthermore, Vujkovic-Cvijin et al. reported that GM is associated with an enhanced systemic IgG response, based on both human epidemiological and animal studies (Vujkovic-Cvijin et al., 2022). Furthermore, differences in GM composition ratios may mediate the activation of plasmacytoid dendritic cells to produce IFN-α and IL-33 and cause IgG4-RD (Yoshikawa et al., 2021).

To date, no studies have examined the causal relationship between GM and serum IgG4 levels in the general population. The GM varies widely according to sex (Yatsunenko et al., 2012; Koliada et al., 2021; Yoon et al., 2021). IgG4-RD has also been reported to show sex-related differences in terms of onset and treatment (Wang et al., 2019). The study hypothesized that gender differences in GM by gender might influence IgG4 levels, as they show gender differences with regard to the development and treatment of IgG4-RD. This study aimed to analyze GM of each male and female patients and use causal inferential methods to determine the relationship between IgG4 levels and GM in the general population.

## 2 Materials and methods

### 2.1 Participants

The participants were 398 residents (191 men and 207 women) aged 40 years or older, of Shika-machi, Hakui-gun, Ishikawa Prefecture, Japan, whose fecal samples were collected in 2019. The following five conditions were excluded from the analysis. 1) participants without measured serum IgG4 levels, 2) patients taking immunosuppressive drugs as described below; Methotrexate and Enbrel, 3) patients taking medications that significantly affect GM as described below; antibiotics, steroids, bowel regulators and antibacterial agents, proton pump inhibitor (PPI), 4) patients suspected cancer and IgG4-related disease, 5) individuals with missing data, 6) patients with inflammatory bowel disease (IBD).

### 2.2 Data collection

Data from the Shika-machi Super Preventive Health Examination, a population survey aimed at establishing preventive methods for lifestyle-related diseases, were used. The survey was conducted between 2019. The four model districts selected from the Shika area were Horimatsu, Higashimasuho, Tsuchida, and Higashiki (Karashima et al., 2018; Nagase et al., 2020).

The Shika-machi Super Preventive Health Checkup data regarding parameters such as age, sex, medical history, medication status, allergy status, and alcohol consumption/smoking status were collected using a questionnaire. The body mass index (BMI) was calculated by dividing the current weight (kg) by the square of the height (m^2^). Venous blood was collected early in the morning after a 12-hour fast. The 24-hour urinary sodium excretion was calculated based on the 24-hour urinary creatinine and sodium excretion values (Nagase et al., 2020). The estimated daily salt intake was calculated using 24-hour urinary sodium excretion.

Immune-related blood samples were measured using the following test kits; IgG4 (IgG4 subclass BS-TIA3 IgG4, MEDICAL & BIOLOGICAL LABORATORIES CO., LTD., Tokyo, Japan); Immunoglobulin G (IgG) (N-assay TIA IgG-SH Nittobo, NITTOBO MEDICAL CO., LTD., Tokyo, Japan); Immunoglobulin E (IgE) (ImmunoCAP Total IgE, THERMO FISHER SCIENTIFIC INC., Waltham, MA, USA); 50% hemolytic unit of complement (CH50) (auto CH50-L eikenII, DENKA COMPANY LIMITED, Tokyo, Japan); Anti-CCP antibody (Stacia MEBLux test CCP, MEDICAL & BIOLOGICAL LABORATORIES CO., LTD., Tokyo, Japan); antinuclear antibody (ANA) (anti-nuclear antibody (ANA) (FA) [FR], FUJIREBIO INC., Tokyo, Japan); Aniti-SS-A/Ro antibody (stacia MEBLux test SS-A, MEDICAL & BIOLOGICAL LABORATORIES CO., LTD., Tokyo, Japan); rheumatoid factor (RF) levels (LZtest ‘eiken’ RF, EIKEN CHEMICAL CO., LTD., Tokyo, Japan).

### 2.3 DNA extraction and next-generation sequencing

Fecal samples were collected using previously described methods (Miyajima et al., 2022) and stored at −80°C until DNA extraction. The processing of fecal samples was carried out in a non-proliferation level 2 (P2) laboratory. The DNA extracted from the GM was processed to identify the 16S rRNA gene sequence using a previously reported next-generation sequencing method (Miyajima et al., 2022).

### 2.4 Microbiome analysis

For microbiome analysis, QIIME2 software was used (Bolyen et al., 2022). Demultiplexed paired-end sequence data were denoised with DADA2, and the Silva 16S rRNA database (release 132) naïve Bayes classifier was used for Amplicon Sequence Variant classification (Quast et al., 2013). Samples with fewer than 5000 sequences were removed from the analysis.

### 2.5 Statistical analysis

Statistical analysis and machine learning were performed using Python (version 3.10.9) (Pedregosa et al., 2011) or R, using R-studio (version 4.2.3, RStudio, Boston, MA, United States).

The clinical information of the participants underwent a normality assessment using the Shapiro-Wilk test. Normally distributed data are expressed as mean ± standard deviation, while non-normally distributed data are presented as median (25^th^–75^th^ percentile). The significance of differences in clinical information between the groups was assessed using Student’s t-test for normally distributed data and the Wilcoxson rank-sum test for non-normally distributed data.

The patients were categorized into two groups, high and low, based on the median values of IgG4. Quade’s non-parametric ANCOVA and Tukey’s HSD test were used to compare the relative proportions of GM between the high and low IgG4 groups. Confounders such as age, sex, BMI, daily salt intake, frequency of alcohol consumption per week, and smoking were adjusted for (Vujkovic-Cvijin et al., 2020). Additionally, the clinical background variables that exhibited significant differences between the high and low IgG4 groups were included as new confounding factors. The significance level for all tests was set at *P* < 0.05. Alpha diversity was evaluated using the Shannon index, with Amplicon Sequence Variant values (Willis, 2019). To assess the beta diversity, non-metric multidimensional scaling analysis with the Bray-Curtis dissimilarity metric from the “vegan” package in R was used, along with permutation multivariate analysis of variance (Dixon, 2003). To identify GM associated with IgG4, linear discriminant analysis effect size (LEfSe) was employed (Segata et al., 2011).

The odds ratios and *P*-values were calculated using the least absolute shrinkage and selection operator logistic regression model (LASSO logistic regression) from the “glmnet” package in R (Tibshirani, 1996). Multicollinearity was assessed using the variance expansion factor (VIF) and only bacterial genera with a VIF smaller than 10 were used in the LASSO analysis. Correlation coefficients and *P*-values were calculated using Spearman’s rank correlation coefficient in R’s “Package ppcor” after adjusting for the variables listed above. The correlation coefficients were plotted using “Package pheatmap”.

The heat maps were visualized as dendrograms using hierarchical clustering, which was based on similarity by correlation coefficient. Bacterial genera that were significantly correlated with IgG4 and one bacterial genus with the closest inter-cluster distance was set up as a new bacterial genus group. The closest bacterial genus was not grouped if it was a population of several bacterial genera.

The direct linear non-Gaussian acyclic model (LiNGAM) model was built using “LiNGAM” in Python (Shimizu et al., 2011). The bacterial genera chosen for LiNGAM algorithm were selected based on their significant associations identified in at least one of the following analyses: ANCOVA, Tukey’s HSD, LEfSe, LASSO, and Correlation analyses. To demonstrate the robustness and consistency of the causal relationships, the occurrence rates and partial regression coefficients of the causal relationships were presented. Unselected bacterial genera were entered exhaustively as noise, and their impact on causality was observed (Mizoguchi et al., 2023).

## 3 Results

### 3.1 Clinical background

Data on GM were procured from the fecal specimens of 234 study participants. The study dismissed 138 samples lacking IgG4 quantification, six individuals under immunosuppressive or gut flora-altering medications, one potential cancer case, and one suspected IgG4-RD case. None of the participants had IBD. In total, 88 patients (46 females and 42 males) participated in the analysis. **Supplementary Figure 1** contains a flowchart on sample selection. **Table 1** elucidates their clinical data. Participants were sorted into high and low categories based on their median serum IgG4 values. The median IgG4 value for all participants was 41.7 mg/dL. By gender, the median values stood at 34.8 mg/dL for females and 57.1 mg/dL for males. Significant discrepancies in BMI, IgG4, IgE, alcohol consumption, and smoking prevalence were observed between genders. **Supplementary Tables 1**–**3** respectively offer a comparative overview of the immunological landscape of high and low IgG4 cohorts of all participants, women and men, respectively.

**Table 1:** Clinical characteristics of study participants categorized by sex.

	all	female	male	*P*-value
n	88	46	42	
Age (years)	62 ± 11	62 ± 10	64 ± 11	0.318
BMI (kg/m2)	23.4 ± 3.0	22.8 ± 3.3	24.1 ± 2.6	0.038
IgG4 (mg/dl)	41.7 (23.8-76.2)	34.8 (21.4-60.1)	57.1 (31.4-81.5)	0.013
IgG (mg/dl)	1347.6 ± 269.8	1355.0 ± 260.6	1339.6 ± 279.3	0.791
IgE (IU/ml)	77.1 (31.2-170.8)	48.2 (23.5-141.3)	137 (47.6-253.3)	0.003
CH50 (U/ml)	43.9 (38.9-48.9)	45.1 (40.3-49.9)	42.2 (38.6-47.7)	0.171
Anti-CCP antibody (U/ml)	0.6 (0.6-0.6)	0.6 (0.6-0.6)	0.6 (0.6-0.6)	0.891
ANA (times)	40 (40-40)	40 (40-40)	40 (40-40)	0.470
Aniti-SS-A/Ro antibody (U/ml)	1 (1-1)	1 (1-1)	1 (1-1)	0.187
RF (IU/ml)	5 (5-8.5)	5.5 (5-10.8)	5 (5-8)	0.229
Allergy (%)	11.4	10.9	11.4	0.880
Alcohol consumption (day/week)	0 (0-4)	0 (0-0.8)	3 (0-7)	<0.001
Salt intake (g/day)	9.2 (8.0-10.6)	9.1 (8.3-10.3)	9.6 (7.6-10.8)	0.875
Smoking (%)	19.3	10.9	27.9	0.036

P values were calculated using covariance analysis (ANCOVA or Quade’s nonparametric ANCOVA). ANCOVA, analysis of covariance; BMI, body mass index; CCP, cyclic citrullinated peptide; IgG4, Immunoglobulin G4; IgG, Immunoglobulin G; IgE, Immunoglobulin E; CH50, 50% hemolytic unit of complement; ANA, antinuclear antibody; RF, rheumatoid factor.

### 3.2 Comparison of gut microbiota composition

**Figure 1:**
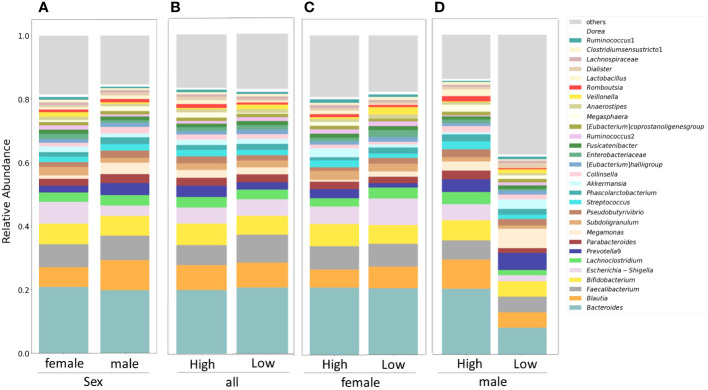
Comparison of relative abundance ratios at the genus level for the top 30 bacterial genera with mean abundance ratios by sex **(A)**. Differences in gut microbiota between female and male groups. Differences in gut microbiota between high and low IgG4 groups in all participants **(B)**, women **(C)** and men **(D)**.

**Figure 1A** displays stacked plots showing the mean relative abundance of the top 30 bacterial genera among women and men. The top 30 bacterial genera accounted for an average of 81% of women and 85% of men. **Figure 1B** demonstrates the mean relative abundance of the top 30 genera in the high IgG4 and low IgG4 groups for all participants. Among female participants, the top 30 bacterial genera accounted for an average of 83% of the high IgG4 group and 83% of the low IgG4 group. When segregated by gender, they constituted 83% for both IgG4 groups among women (**Figure 1C**), while for men, they accounted for 82% and 62% in the high and low IgG4 groups, respectively (**Figure 1D**).

**Figure 2:**
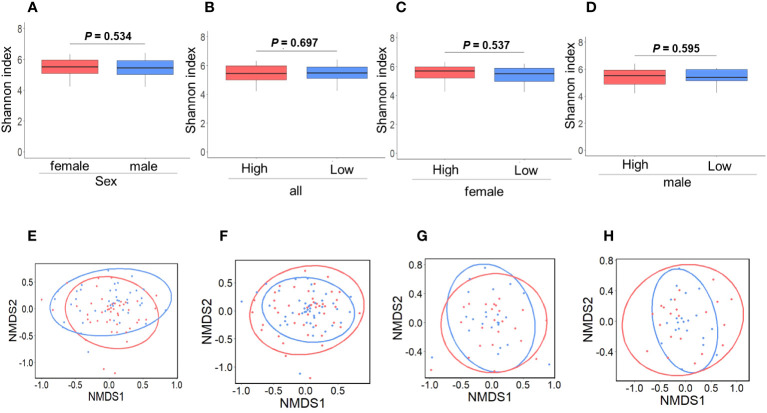
Comparison of gut microbiota diversity. Comparison between female and male groups. a-diversity (**A**; *P* = 0.534), β-diversity (**E**; *P* = 0.224) (*P* = 0.534). Red and blue indicate females and males, respectively. Comparison between the high and low IgG4 groups in all participants. a-diversity (**B**; *P* = 0.697), β-diversity (**F**; *P* = 0.706). Comparison between the high and low IgG4 groups in women. a-diversity (**C**; *P* = 0.537), β-diversity (**G**; *P* = 0.854). Comparison between men in the high and low IgG4 groups a-diversity (**D**; *P* = 0.224), β-diversity (**H**; *P* = 0.623),. Red indicates the high IgG4 group and blue indicates the low IgG4 group.

**Figures 2A–D** and **2E–H** depict the alpha and beta diversities, respectively, and the analyses revealed no significant disparities in gut GM diversity between sexes (**Figures 2A, E**) or among the high and low IgG4 groups (**Figures 2B–D, F–H**).

**Figure 3:**
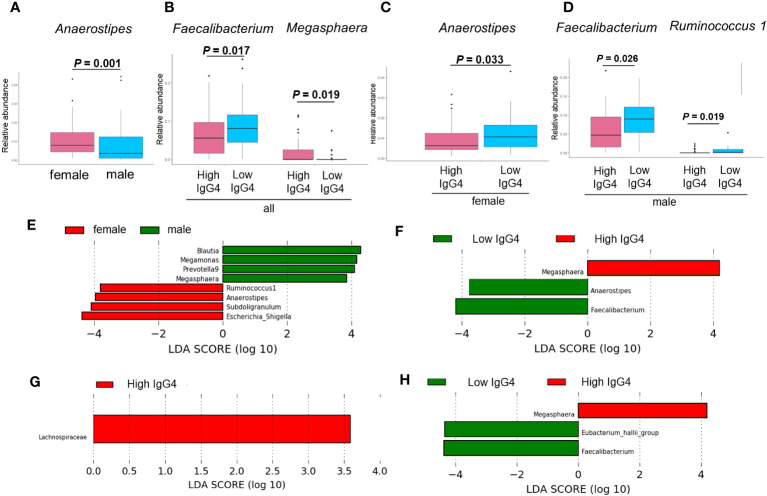
Comparison of gut microbiota between groups. Comparison between men and women by ANCOVA **(A)**. Bacterial genera showing significant differences between high and low IgG4 groups in all participants **(B)**, women **(C)** and men **(D)**. Comparison between men and women by LEfSe **(E)**. Bacterial genera with Linear discriminant analysis (LDA) score of 2 or higher between high and low IgG4 groups in all participants **(F)** females **(G)**, and males.

**Figure 3** demonstrates the significant differences in the presence of specific bacterial genera between IgG4 groups and sexes: *Anaerostipes* were more prevalent in women than men (**Figure 3A**). In all participants, the proportion of *Faecalibacterium* present in the High IgG4 group was significantly lower than in the Low IgG4 group, and the proportion of *Megasphaera* present in the High IgG4 group was significantly higher than in the Low IgG4 group (**Figure 3B**). Women exhibited a significantly lower representation of *Anaerostipes* in the High IgG4 group (**Figure 3C**), while men in the High IgG4 group had significantly diminished proportions of *Faecalibacterium* and *Ruminococcus.*1 (**Figure 3D**).

LEfSe revealed that the relative abundance of *Blautia, Megamonas, Prevotella* 9*, Megasphaera, Ruminococcus.*1*, Anaerostipes, Subdoligranulum, Escherichia-Shigella* has difference among women and men (**Figure 3E**). In all patients, the relative abundance of *Megasphaera* was higher in the high IgG4 group, and the relative abundances of *Anaerostipes* and *Faecalibacterium* were lower in the low IgG4 group (**Figure 3H**). In females, that of *Lachnospiraceae* was higher in the high IgG4 group (**Figure 3G**). In males, that of *Megasphaera* was higher in the high IgG4 group, and the relative abundance of *Faecalibacterium* and *[Eubacterium] hallii* group was lower in the low IgG4 group (**Figure 3H**).

### 3.3 LASSO model for predicting the classification of high and low IgG4 groups

**Figure 4:**
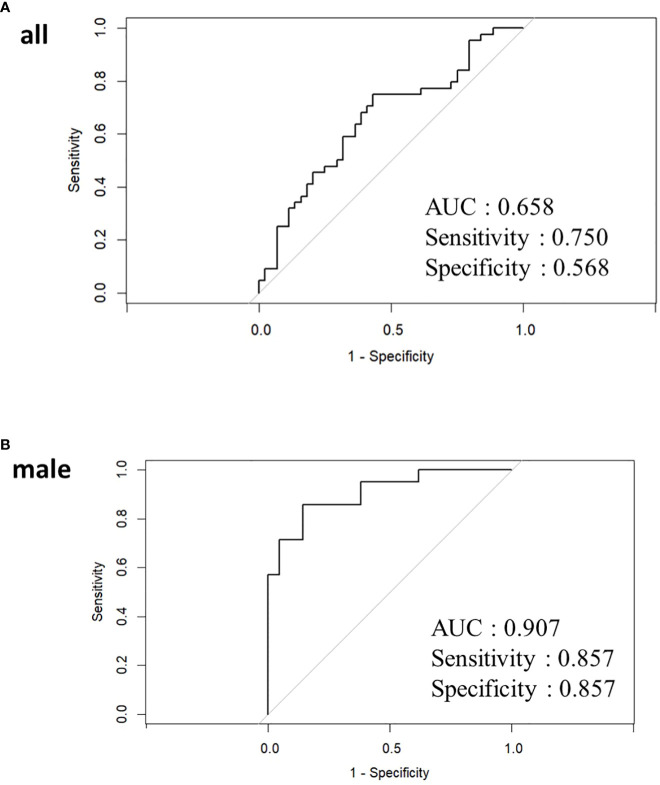
Receiver operating characteristic curve curves for LASSO analysis in all participants **(A)** and male **(B)**. In women, the LASSO model could not be applied because Parabacteroides was the only bacterial genus with a dispersal expansion coefficient below 10.

A predictive model for classifying high/low IgG4 groups was developed using GM data in a LASSO logistic regression model. Thirteen bacterial genera (*Blautia, Bifidobacterrium, Subdoligranulum, Streptococcus, Collinsella, Enterobacteriaceae, Fusicatenibacter,. [Eubacterium] hallii* group, *Anaerostipes, Veillonella, Romboutsia, Lactobacillus, and Ruminococcus.*1) had VIF <10 in all participants. In LASSO in all participants, none of the 13 bacterial genera had statistically significant odds ratios. The classification prediction model in all participants was the area under the receiver operating characteristic curve showed 0.658, sensitivity 0.750, and specificity 0.568 (**Figure 4A**).

Twelve bacterial genera (*Blautia*, *Bifidobacterium*, *Parabacteroides*, *Collinsella*, *[Eubacterium] hallii* group, *Fusicatenibacter*, *[Eubacterium] coprostanoligenes* group, *Megasphaera*, *Anaerostipes*, *Veillonella*, and *Romboutsia*) had VIF <10 in male. Of the 12 bacterial genera, only *Romboutsia* was statistically significant with an odds ratio of 2.696 (95% confidence interval 1.031-7.050, P=0.043) (**Supplementary Table 4**). The classification prediction model in males was the area under the receiver operating characteristic curve showed 0.907, sensitivity 0.857, and specificity 0.857 (**Figure 4B**). Only Parabacteroides had a VIF of less than 10, and other bacterial genera showed multicollinearity in women. For Parabacteroides alone, no predictive model could be built by LASSO and no ROC curve could be drawn.

### 3.4 Correlation and causality diagram between IgG4 and GM

**Figure 5:**
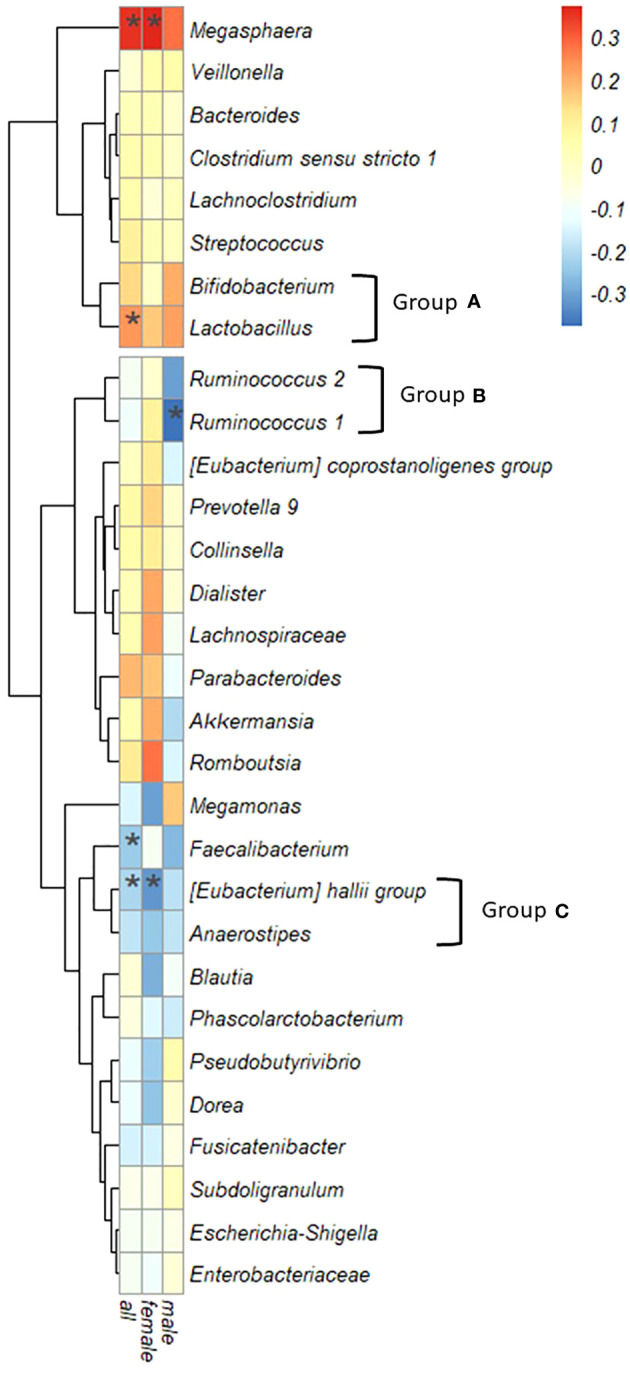
Correlation between serum IgG4 levels and bacterial genera. Spearman’s correlation coefficient, the color intensity of the heatmap is defined by Spearman’s correlation coefficient. Hierarchical cluster analysis allowed relationships between bacteria to be visualized by dendrograms based on correlations with IgG4. (*: *P* < 0.05).

**Figure 5** illustrates the correlation between IgG4 levels and the top 30 intestinal bacterial species, considering the relative abundance. *Megasphaera* and *Lactobacillus* displayed significant positive correlations with IgG4 levels in the entire cohort, while *Faecalibacterium* and *[Eubacterium] hallii* group exhibited significant negative correlations. In women, IgG4 levels were correlated positively with *Megasphaera* and negatively with *[Eubacterium] hallii* group. In men, a negative correlation was observed with *Ruminococcus*.1.

Based on the similarity of correlation coefficients between IgG4 values and bacterial genera visualized in a dendrogram, three groups were redefined: Group A encompassed *Bifidobacterium* and *Lactobacillus*; Group B incorporated *Ruminococcus*.1 and *Ruminococcus*.2; and Group C comprised the *[Eubacterium] hallii* group and *Anaerostipes.*

### 3.5 Causal inference by LiNGAM model

Causal inference with the direct LiNGAM model using bacterial genera were significantly associated with IgG4 in ANCOVA, Tukey’s HSD, LefSe, LASSO, and Correlation analyses.

**Supplementary Figure 2** shows the results of LiNGAM with bacterial genera and IgG4 listed in **Supplementary Table 5**. No causal relationships were estimated between bacterial genera and IgG4.

**Figure 6:**
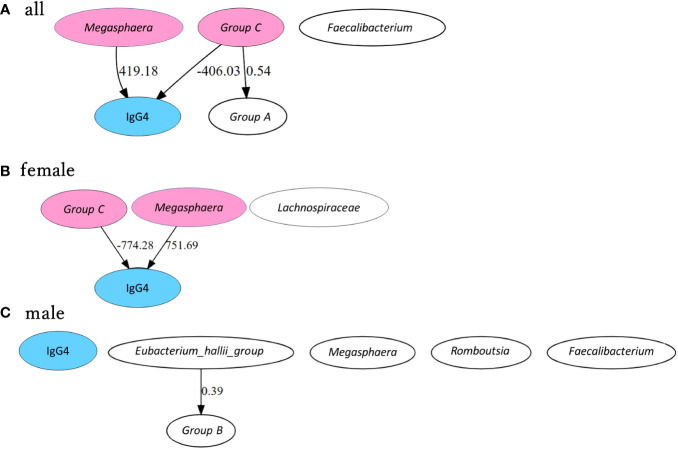
Causal inference between IgG4 levels and bacterial genus by GM group. Causal inference results are presented separately for all participants **(A)**, female participants **(B)** and male participants **(C)**. The arrows indicate the direction of causality between two connected indicators. Values are partial regression coefficients. Groups A, B, and C were grouped based on phylogenetic trees according to the correlation between bacterial genera and IgG4. Group A includes Bifidobacterium + Lactobacillus, Group B includes Ruminococcus.1 + Ruminococcus.2 and Group C includes [Eubacterium] hallii group + Anaerostipes. IgG4 is highlighted in blue, and bacterial genera and bacterial groups presumed to be causally related in each group are highlighted in pink.

**Figure 6** shows the estimated causal relationship with IgG4, including the redefined bacterial groups. In all participants and women, an increase in *Megasphaera* was also associated with an increase in serum IgG4 levels, while an increase in group C (*[Eubacterium] hallii* group and *Anaerostipes*) was associated with a decrease in serum IgG4 levels. In the robustness analysis of causal results, a causal direction from *Megasphaera* to IgG4 was detected in 82.6% of cases (partial regression coefficient 385.0 ± 45.9) and from group C to IgG4 in 78.3% of cases (partial regression coefficient -359.4 ± 62.9) in all participants. A causal direction from *Megasphaera* to IgG4 was detected in 81.8% of cases (partial regression coefficient 673.7 ± 73.7) and from group C to IgG4 in 59.1% of cases (partial regression coefficient -710.0 ± 84.3) in women. In contrast, no causal relationship between bacterial genus and IgG4 could be inferred in males.

## 4 Discussion

Women had a significantly higher proportion of *Anaerostipes* than men in the general population. Causal inference in women showed that *Megasphaera* increased IgG4 levels, while the groups including *[Eubacterium] hallii* group and *Anaerostipes* decreased IgG4 levels. In men, *Megasphaera*, *[Eubacterium] hallii* group, *Faecalibacterium*, *Ruminococcus.*1, and *Romboutsia* were important bacterial genera for classifying high IgG4 groups and low IgG4 groups. No bacterial genera presented a causal relationship with serum IgG4 levels in men. Serum IgG4 levels may be associated with changes in the gut bacterial genera.

Several studies have reported on the association between IgG4-RD and GM (Wang et al., 2019; Liu et al., 2021; Plichta et al., 2021). Yoshikawa et al. reported that abnormalities in the GM mediate the activation of plasmacytoid dendritic cells, which produces IFN-α and IL-33, causing experimental autoimmune pancreatitis and IgG4-RD (Wang et al., 2019). Liu et al. also found that in IgG4-related sclerosing cholangitis, marked depletion of *Blautia* and elevated succinate may be responsible for hepatitis (Liu et al., 2021). These reports reinforce the relationship between GM and IgG4-RD development. However, the bacteria they reported were not entirely consistent with the bacterial genera in this study that identified the relationship.

*Megasphaer*a is a genus of anaerobic bacteria that metabolizes short-chain fatty acids such as acetic, butyric, and isobutyric acid (Jeon et al., 2017). In the Japanese population, *Megasphaera* is more prevalent in males than in females (Hatayama et al., 2023). In our study, the LEfSe analysis also identified the proportion of *Megasphaera* composition as a significant bacterial flora characteristic distinguishing between men and women. Furusawa et al. reported that butyric acid produced by microorganisms induces the differentiation of regulatory T cells, which play a role in suppressing allergic reactions (Furusawa et al., 2013). Moreover, Dong et al. reported an association between *Megasphaera* and IgA nephropathy, in which IgA, a type of immunoglobulin, is deposited in the glomeruli (Dong et al., 2020).

The *[Eubacterium] hallii* group and *Anaerostipes* were categorized as closely related based on the similarity of their correlations with IgG4. Shetty et al. have demonstrated the close relationship between *[Eubacterium] hallii* group and *Anaerostipes* using a multifaceted approach and have recommended their reclassification (Shetty et al., 2018). They both can convert lactic acid to butyric acid, a short chain fatty acid, and may have similar functional roles in the gut (Belenguer et al., 2006). Additionally, *[Eubacterium] hallii* group was found to be enriched in the GM of patients with chronic inflammatory demyelinating polyneuritis, a chronic autoimmune disease affecting the peripheral nerves, compared to healthy subjects (Svačina et al., 2023). Furthermore, the abundance of *Anaerostipes* was found to differ significantly between the immune antibody-positive and -negative groups in patients with immune antibody-positive-related repeated miscarriages (Jin et al., 2020).

These findings suggest a close relationship between IgG4-RD, immune diseases, and GM development and pathogenesis, which may be mediated by GM-derived short-chain fatty acids. Fatty acids play a crucial role in the differentiation of Th0 cells into TH2 cells, which are responsible for the release of interleukin-4 (IL-4) (Asarat et al., 2015). IL-4, IL-10, IL-21, IL-13, and B cell-activating factors have been found to be correlated with IgG4 production (Maehara et al., 2012; Tanaka et al., 2012; Watanabe et al., 2013; Akiyama et al., 2018; Maehara et al., 2018; Lanzillotta et al., 2020). Therefore, it is plausible that short-chain fatty acids may influence IgG4 production through their impact on inflammatory cytokines.

We have newly redefined bacterial groups based on the similarity of correlations of bacterial genera to IgG4. As gut bacteria are thought to interact with each other to create a favorable habitat, it is necessary to not only find a relationship between one bacterial genus and IgG4 but also to evaluate bacterial genera with similarities to each other. Both *Lactobacilli* and *Bifidobacteria* are non-spore-forming, gram-positive, lactic acid-producing bacteria. Despite some common properties, *Lactobacilli* and *Bifidobacteria* belong to two taxonomically distinct groups: the genus *Lactobacillus* in the phylum *Firmicutes* and the genus *Bifidobacterium* in the phylum *Actinobacteria*, respectively (Vlasova et al., 2016). *Ruminococcus*.1 and *Ruminococcus*.2 are also considered part of the phylum *Bacillota*, the *Bacillota* web, and the order *Eubacteriales* and are classified as *Ruminococcus*.1 and *Ruminococcus*.2 in the SILVA database (Bolyen et al., 2022). These combinations have been reported to show high genetic similarity by comparison of 16S rRNA sequences (Vlasova et al., 2016; Henderson et al., 2019), therefore, it is reasonable to redefine them as a group. Direct LiNGAM inferred a causal relationship between bacterial genera and bacterial groups, but the complexity of gut-bacterial interactions is high and many aspects need to be clarified and require further research.

This study has several limitations that should be considered. Firstly, although the three bacterial genera *Megasphaera*, *[Eubacterium] hallii* group, and *Anaerostipes* identified in this study are known to produce butyrate, it cannot be definitively concluded that these bacteria are causally related to IgG4. Other SCFA-producing bacteria may also be involved, and the underlying mechanisms of action of these bacteria need further investigation. Secondly, lifestyle factors involved in IgG4-RD may not be adequately considered. Some researchers have argued that lifestyle habits, such as smoking, contribute to the development of IgG4-RD (Wallwork et al., 2021; Tsuji et al., 2023). The Direct LiNGAM model cannot correctly analyze for unobserved confounders. It cannot be ruled out that lifestyle differences based on gender may be a confounding factor for gut bacteria and high IgG4 levels. Further studies should therefore be conducted, e.g. in animal models that are unaffected by gender differences in lifestyle. Finally, it should be noted that the participants were not patients with IgG4-RD. However, no report has attempted to identify a causal relationship between GM and serum IgG4 levels in the general population, which underlines the importance of this study. Further studies are needed to clarify the influence of GM on IgG4-RD development.

In conclusion, *Megasphaera*, *[Eubacterium] hallii* group, and *Anaerostipes* were identified as bacterial species that potentially have a causal relationship with IgG4 levels in women. The fact that the impact of the GM on IgG4 levels differs according to gender is a novel and interesting finding. Particularly, the following two points should be considered with caution. (i) the results revealed in the present study, in which the proportion of *Anaerostipes* present in a suspected causal role in reducing IgG4 was significantly higher in women, and (ii) the previously reported fact that women have a lower incidence of IgG4-RD than men. These results may lead to a new hypothesis that women are less likely to develop IgG4-RD than men due to the abundance of *Anaerostipes* in the gut. To elucidate the pathogenesis of IgG4-RD of unknown cause, the metabolites derived from gut bacteria that regulate serum IgG4 levels need to be investigated in detail in the future.

## Data availability statement

The raw data of the sequencing was registered at DNA Data Bank of Japan (DDBJ) (Number DRA016467). **Supplementary Table 6** listed the BioSAMPLE IDs analyzed in the study.

## Ethics statement

The studies involving humans were approved by the ethics committee for Human Studies at Kanazawa University Hospital. The studies were conducted in accordance with the local legislation and institutional requirements. The participants provided their written informed consent to participate in this study.

## Author contributions

AK: Conceptualization, Data curation, Project administration, Validation, Visualization, Writing – original draft, Writing – review & editing. SK: Conceptualization, Funding acquisition, Methodology, Project administration, Supervision, Writing – original draft, Writing – review & editing. KoO: Methodology, Validation, Writing – review & editing, Data curation. YM: Data curation, Investigation, Methodology, Validation, Writing – review & editing. KaO: Methodology, Writing – review & editing. RM: Data curation, Methodology, Validation, Writing – review & editing. YI: Methodology, Writing – review & editing. SH: Investigation, Writing – review & editing. IM: Investigation, Writing – review & editing. HF: Investigation, Writing – review & editing. MK: Investigation, Writing – review & editing. HT: Data curation, Writing – review & editing. AH: Investigation, Writing – review & editing. HN: Writing – review & editing, Investigation. SO: Funding acquisition, Investigation, Supervision, Writing – review & editing.

## References

[B1] Akiyama M. Yasuoka H. Yoshimoto K. Takeuchi T. (2018). Interleukin-4 contributes to the shift of balance of IgG subclasses toward IgG4 in IgG4-related disease. Cytokine 110, 416–419. doi: 10.1016/j.cyto.2018.05.009 29861381

[B2] Asarat M. Apostolopoulos V. Vasiljevic T. Donkor O. (2015). Short-chain fatty acids produced by synbiotic mixtures in skim milk differentially regulate proliferation and cytokine production in peripheral blood mononuclear cells. Int. Food. Sci. Nutr. 66 (7), 755–765. doi: 10.3109/09637486.2015.1088935 26398897

[B3] Belenguer A. Duncan S. H. Calder A. G. Holtrop G. Louis P. Flint H. J. (2006). Two routes of metabolic cross-feeding between Bifidobacterium adolescentis and butyrate-producing anaerobes from the human gut. Appl. Environ. Microbiol. 72 (5), 3593–3599. doi: 10.1128/AEM.72.5.3593-3599.2006 16672507 PMC1472403

[B4] Bolyen E. Rideout J. R. Dillon M. R. Bokulich N. A. Abnet C. C. Al-Ghalith G. A.. (2022). QIIME 2 docs. Available at: https://docs.qiime2.org/2022.2/.

[B5] Chen Y. Bai J. Wu D. (2021). Role and mechanism of gut microbiota in human disease. Front. Cell. Infect. Microbiol. 11. doi: 10.3389/fcimb.2021.625913 PMC8010197 33816335

[B6] Clemente J. C. Ursell L. K. Parfrey L. W. (2012). The impact of the gut microbiota on human health: an integrative view. Cell 148 (6), 1258–1270. doi: 10.1016/j.cell.2012.01.035 22424233 PMC5050011

[B7] Davies A. M. Sutton B. J. (2015). Human IgG4: a structural perspective. Immunol. Rev. 268, 139–159. doi: 10.1111/imr.12349 26497518 PMC4670484

[B8] Dixon P. (2003). VEGAN, a package of R functions for community ecology. J. Veg Sci. 14, 927–930. doi: 10.1111/j.1654-1103.2003.tb02228.x

[B9] Dong R. Bai M. Zhao J. Wang D. Ning X. Sun S. (2020). A comparative study of the gut microbiota associated with immunoglobulin A nephropathy and membranous nephropathy. Front. Cell. Infection Microbiol. 10, 557368. doi: 10.3389/fcimb.2020.557368 PMC7606180 33194798

[B10] Furusawa Y. Obata Y. Fukuda S. Endo T. A. Nakato G. Takahashi D.. (2013). Commensal microbe-derived butyrate induces the differentiation of colonic regulatory T cells. Nature 504, 446–450. doi: 10.1038/nature12721 24226770

[B11] Hatayama K. Sakamoto M. Fukushima K. Ishii M. Kawabata H. (2023). Sex differences in intestinal microbiota and their association with some diseases in a Japanese population observed by analysis using a large dataset. Biomedicines 11 (2), 376. doi: 10.3390/biomedicines11020376 36830915 PMC9953495

[B12] Henderson G. Yilmaz P. Kumar S. Forster R. J. Kelly W. J. Leahy S. C.. (2019). Improved taxonomic assignment of rumen bacterial 16S rRNA sequences using a revised SILVA taxonomic framework. PeerJ 7, e6496. doi: 10.7717/peerj.6496 30863673 PMC6407505

[B13] Jeon B. S. Kim B. C. Kim H. Y. Lee S. Y. Kim H. J. Kim J. S. (2017). Megasphaera hexanoica sp. nov., a medium-chain carboxylic acid-producing bacterium isolated from a cow rumen. Int. J. Syst. Evol. Microbiol. 67 (7), 2114–2120. doi: 10.1099/ijsem.0.001888 28742009

[B14] Jiao Y. Wu L. Huntington N. D. (2020). Crosstalk between gut microbiota and innate immunity and its implication in autoimmune diseases. Front. Immunol. 11. doi: 10.3389/fimmu.2020.00282 PMC7047319 32153586

[B15] Jin M. Qiu L. Wang L. Zhou Y. Wang T. Ying H.. (2020). Changes in gut microorganism in patients with positive immune antibody-associated recurrent abortion. BioMed. Res. Int. 2020, 4673250. doi: 10.1155/2020/4673250 33015167 PMC7520699

[B16] Karashima S. Kometani M. Tsujiguchi H. Asakura H. Nakano S. Usukura M.. (2018). Prevalence of primary aldosteronism without hypertension in the general population: results in Shika study. Clin. Exp. Hypertens. 40, 118–125. doi: 10.1080/10641963.2017.1339072 28723305

[B17] Koliada A. Syzenko G. Moseiko V. Budovska L. Puchkov K. Perederiy V.. (2021). Sex differences in the phylum-level human gut microbiota composition. BMC Microbiol. 21 (1), 131. doi: 10.1186/s12866-021-02139-w 33931023 PMC8088078

[B18] Lanzillotta M. Mancuso G. Della-Torre E. (2020). Advances in the diagnosis and management of IgG4 related disease. BMJ 369, m1067. doi: 10.1136/bmj.m1067 32546500

[B19] Liu Q. Li B. Li Y. Wei Y. Huang B. Liang J.. (2021). Altered faecal microbiome and metabolome in IgG4-related sclerosing cholangitis and primary sclerosing cholangitis. Gut 71 (5), 899–909. doi: 10.1136/gutjnl-2020-323565 34035120

[B20] Lu H. Zhang Y. Zhang J. (2021). Differences in clinical characteristics of IgG4-related disease across age groups: a prospective study of 737 patients. Rheumatol. (Oxford) 60 (6), 2635–2646. doi: 10.1093/rheumatology/keab090 33211878

[B21] Maehara T. Mattoo H. Mahajan V. S. Murphy S. J. Yuen G. J. Ishiguro N.. (2018). The expansion in lymphoid organs of IL-4+ BATF+ T follicular helper cells is linked to IgG4 class switching *in vivo* . Life Sci. Alliance 1 (1), e201800050. doi: 10.26508/lsa.201800050 29984361 PMC6034714

[B22] Maehara T. Moriyama M. Nakashima H. Miyake K. Hayashida J. N. Tanaka A.. (2012). Interleukin-21 contributes to germinal centre formation and immunoglobulin G4 production in IgG4-related dacryoadenitis and sialoadenitis, so-called Mikulicz’s disease. Ann. Rheumatic Dis. 71 (12), 2011–2019. doi: 10.1136/annrheumdis-2012-201477 22753386

[B23] Maslinska M. Trojanowska M. Radzikowska U. (2022). The role of igG4 in autoimmunity and rheumatic diseases. Front. Immunol. 12. doi: 10.3389/fimmu.2021.787422 PMC8821096 35145508

[B24] Miyajima Y. Karashima S. Ogai K. Taniguchi K. Ogura K. Kawakami M.. (2022). Impact of gut microbiome on dyslipidemia in Japanese adults: Assessment of the Shika-machi super preventive health examination results for causal inference. Front. Cell Infect. Microbiol. 12. doi: 10.3389/fcimb.2022.908997 PMC9479221 36118024

[B25] Mizoguchi R. Karashima S. Miyajima Y. Ogura K. Kometani M. Aono D.. (2023). Impact of gut microbiome on the renin-aldosterone system: Shika-machi Super Preventive Health Examination results. Hypertens. Res. doi: 10.1038/s41440-023-01334-7 37280260

[B26] Nagase S. Karashima S. Tsujiguchi H. Tsuboi H. Miyagi S. Kometani M.. (2020). Impact of gut microbiome on hypertensive patients with low-salt intake: Shika study results. Front. Med. 7. doi: 10.3389/fmed.2020.00475 PMC7492604 32984370

[B27] Pedregosa F. Varoquaux G. Gramfort A. Michel V. Thirion B. Grisel O.. (2011). Scikit-learn: machine learning in python. J. Mach. Learn Res. 12, 2825–2830.

[B28] Plichta D. R. Somani J. Pichaud M. Wallace Z. S. Fernandes A. D. Perugino C. A.. (2021). Congruent microbiome signatures in fibrosis-prone autoimmune diseases: IgG4-related disease and systemic sclerosis. Genome Med. 13 (1), 35. doi: 10.1186/s13073-021-00853-7 33648559 PMC7919092

[B29] Quast C. Pruesse E. Yilmaz P. Gerken J. Schweer T. Yarza P.. (2013). The SILVA ribosomal RNA gene database project: Improved data processing and web-based tools. Nucleic Acids Res. 41, D590–D596. doi: 10.1093/nar/gks1219 23193283 PMC3531112

[B30] Segata N. Izard J. Waldron L. Gevers D. Miropolsky L. Garrett W. S.. (2011). Metagenomic biomarker discovery and explanation. Genome Biol. 12, R60. doi: 10.1186/gb-2011-12-6-r60 21702898 PMC3218848

[B31] Shetty S. A. Marathe N. P. Lanjekar V. B. Ranade D. R. (2018). Reclassification of Eubacterium hallii as Anaerobutyricum hallii gen. nov., comb. nov., and description of Anaerobutyricum soehngenii sp. nov., a butyrate and propionate-producing bacterium from infant faeces. Int. J. Syst. Evol. Microbiol. 68 (12), 3741–3745. doi: 10.1099/ijsem.0.003041 30351260

[B32] Shimizu S. Inazumi T. Sogawa Y. Hyvarinen A. Kawahara Y. Washio T.. (2011). A direct method for learning a linear non-Gaussian structural equation model. J. Mach. Learn Res. 12, 1225–1248.

[B33] Svačina M. K. R. Hall J. W. Pritchard J. F. Pritchard T. Ajroud-Driss S. Wolfe G. I.. (2023). The gut microbiome in intravenous immunoglobulin-treated chronic inflammatory demyelinating polyneuropathy. Eur. J. Neurol. 00, 1–6. doi: 10.1111/ene.15679 36651357

[B34] Tanaka A. Moriyama M. Nakashima H. Miyake K. Hayashida J. N. Maehara T.. (2012). Th2 and regulatory immune reactions contribute to IgG4 production and the initiation of Mikulicz disease. Arthritis Rheumatism 64 (1), 254–263. doi: 10.1002/art.33320 21898360

[B35] Tibshirani R. (1996). Regression shrinkage and selection *via* the lasso. J. R Stat. Soc 58, 267–288. doi: 10.1111/j.2517-6161.1996.tb02080.x

[B36] Tsuji Y. Koga T. Nonaka F. Nobusue K. Kawashiri S. Y. Yamanashi H.. (2023). Identification of risk factors for elevated serum IgG4 levels in subjects in a large-scale health checkup cohort study. Front. Immunol. 14. doi: 10.3389/fimmu.2023.1124417 PMC10031005 36969256

[B37] Umehara H. Okazaki K. Masaki Y. (2021). The 2020 revised comprehensive diagnostic (RCD) criteria for IgG4-RD. Mod. Rheumatol. 31 (3), 529–533. doi: 10.1080/14397595.2021.1896283 33274670

[B38] Vlasova A. N. Kandasamy S. Chattha K. S. Rajashekara G. Saif L. J. (2016). Comparison of probiotic lactobacilli and bifidobacteria effects, immune responses and rotavirus vaccines and infection in different host species. Vet. Immunol. Immunopathol. 172, 72–84. doi: 10.1016/j.vetimm.2016.01.003 26809484 PMC4818210

[B39] Vujkovic-Cvijin I. Sklar J. Jiang L. Natarajan L. Knight R. Belkaid Y. (2020). Host variables confound gut microbiota studies of human disease. Nature 587, 448–454. doi: 10.1038/s41586-020-2881-9 33149306 PMC7677204

[B40] Vujkovic-Cvijin I. Swainson L. A. Chu S. N. Ortiz A. M. Santee C. A. Petriello A.. (2022). The systemic anti-microbiota IgG repertoire can identify gut bacteria that translocate across gut barrier surfaces. Sci. Transl. Med. 14 (658), abl3927. doi: 10.1126/scitranslmed.abl3927 PMC9741845 35976997

[B41] Wallace Z. S. Naden R. P. Chari S. Choi H. Della-Torre E. Dicaire J. F.. (2020). The 2019 american college of rheumatology/European league against rheumatism classification criteria for igG4-related disease. Arthritis Rheumatol. 72 (1), 7–19. doi: 10.1002/art.41120 31793250

[B42] Wallwork R. Perugino C. A. Fu X. Harkness T. Zhang Y. Choi H. K.. (2021). The association of smoking with immunoglobulin G4-related disease: a case-control study. Rheumatology 60 (11), 5310–5317. doi: 10.1093/rheumatology/keab172 33751033 PMC8783539

[B43] Wang L. Zhang P. Lin W. Hu W. Chen H. Wei L.. (2019). Sex disparities in clinical characteristics and prognosis of immunoglobulin G4–related disease: a prospective study of 403 patients. Rheumatology 58 (5), 820–830. doi: 10.1093/rheumatology/key397 30561747

[B44] Wastyk H. C. Jakiela B. Sikora M. Osmenda G. Tykocinski G. Skórka K.. (2021). Gut-microbiota-targeted diets modulate human immune status. Cell 184 (16), 4137–4153.e14. doi: 10.1016/j.cell.2021.06.019 34256014 PMC9020749

[B45] Watanabe T. Yamashita K. Fujikawa S. Sakurai T. Kudo M. Shiokawa M.. (2013). Toll-like receptor activation in basophils contributes to the development of IgG4-related disease. J. Gastroenterol. 48 (2), 247–253. doi: 10.1007/s00535-012-0626-8 22744834

[B46] Willis A. D. (2019). Rarefaction, alpha diversity, and statistics. Front. Microbiol. 10. doi: 10.3389/fmicb.2019.02407 PMC6819366 31708888

[B47] Xu Q. Song P. Wang L. Zhang Y. Cheng G. Qian F. (2022). Causal relationship between gut microbiota and autoimmune diseases: A two-sample mendelian randomization study. Front. Immunol. 12, 746998. doi: 10.3389/fimmu.2021.746998 35140703 PMC8819003

[B48] Yatsunenko T. Rey F. E. Manary M. J. (2012). Human gut microbiome viewed across age and geography. Nature 486, 222–227. doi: 10.1038/nature11053 22699611 PMC3376388

[B49] Yoon K. Kim N. Shim M. (2021). Roles of sex hormones and gender in the gut microbiota. J. Neurogastroenterol. Motil. 27 (3), 314–325. doi: 10.5056/jnm20128 33762473 PMC8266488

[B50] Yoshikawa T. Watanabe T. Kamata K. Hara A. Minaga K. Kudo M. (2021). Intestinal dysbiosis and autoimmune pancreatitis. Front. Immunol. 12. doi: 10.3389/fimmu.2021.621532 PMC8021793 33833754

