# Supplementary figures and images for: Impact of gut microbiome on serum IgG4 levels in the general population: Shika-machi super preventive health examination results
Source: Front Cell Infect Microbiol. 2023 Oct 16;13:1272398. doi: 10.3389/fcimb.2023.1272398 (PMC10613983; doi:10.3389/fcimb.2023.1272398)

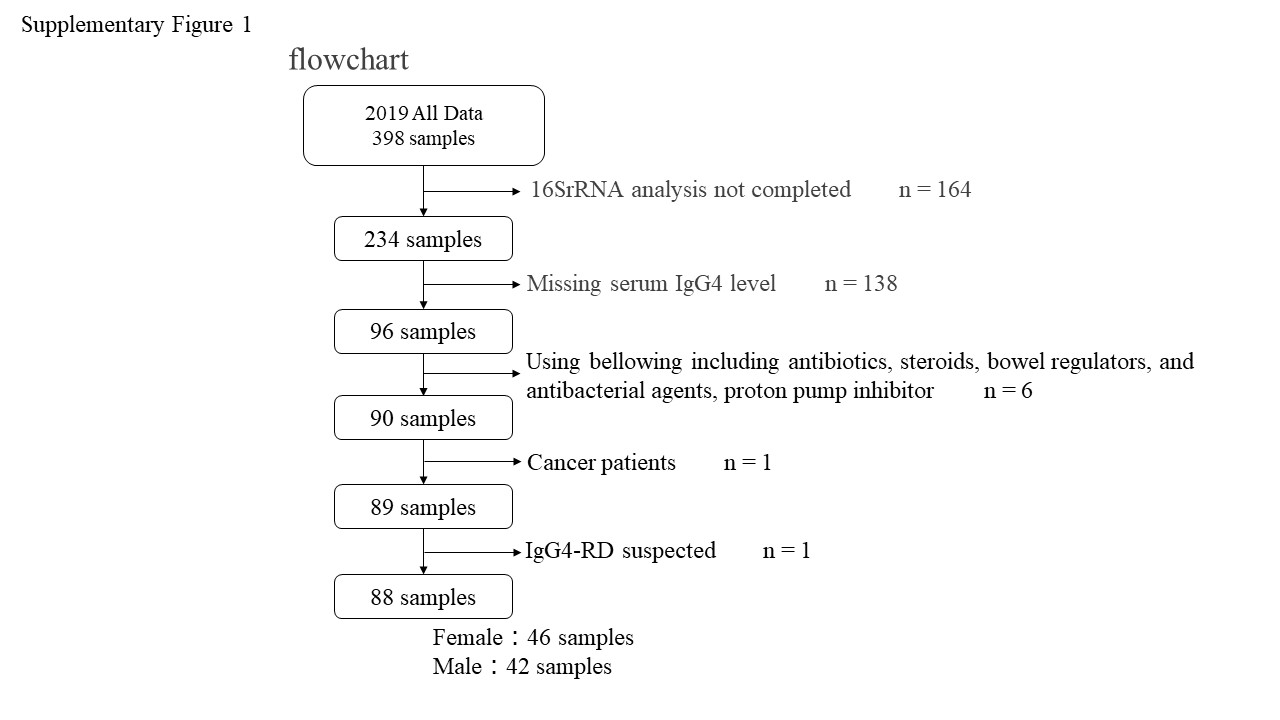

Supplement: Supplementary file 1 [file Image_1.jpeg]

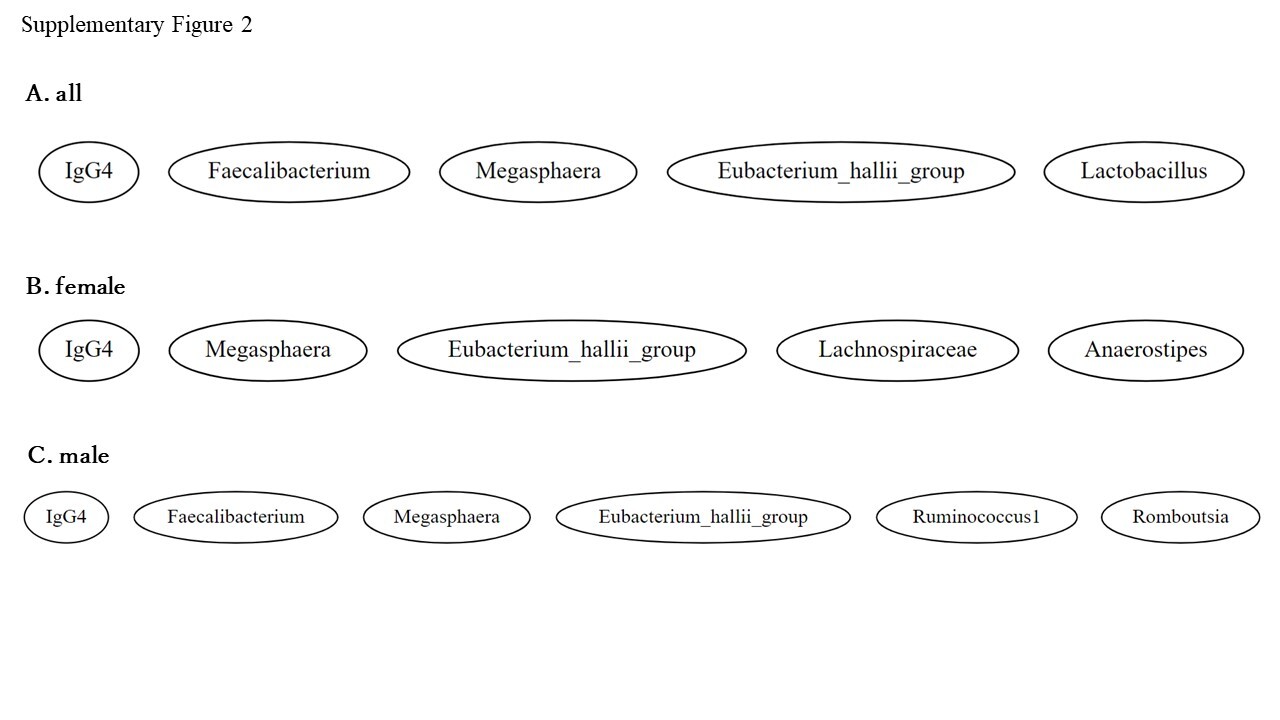

Supplement: Supplementary file 2 [file Image_2.jpeg]
