# Supplementary material for: Impact of gut microbiome on serum IgG4 levels in the general population: Shika-machi super preventive health examination results
Source: Front Cell Infect Microbiol. 2023 Oct 16;13:1272398. doi: 10.3389/fcimb.2023.1272398 (PMC10613983; doi:10.3389/fcimb.2023.1272398)
Supplement: Supplementary file 3 [file DataSheet_1.pdf]

# Impact of Gut Microbiome on serum IgG4 levels in the general population: Shika-machi super preventive health examination results

**Aoi Koshida[1], Shigehiro Karashima[2,3]*, Kohei Ogura[1], Yuna Miyajima[4], Kazuhiro Ogai[5], Ren Mizoguchi[3], Yasuo Ikagawa[1], Satoshi Hara[6], Ichiro Mizushima[6], Hiroshi Fujii[6], Mitsuhiro Kawano[6], Hiromasa Tsujiguchi[7], Akinori Hara[7], Hiroyuki Nakamura[7], Shigefumi Okamoto[1,4]***

[1] Institute for Frontier Science Initiative, Kanazawa University, Kanazawa, Japan

[2] Institute of Liberal Arts and Science, Kanazawa University, Kanazawa, Japan

[3] Department of Health Promotion and Medicine of the Future, Kanazawa University, Kanazawa, Japan

[4] Department of Clinical Laboratory Science, Faculty of Health Sciences, Institute of Medical, Pharmaceutical and Health Sciences, Kanazawa University, Kanazawa, Japan

[5] Department of Bio-engineering Nursing, Graduate School of Nursing, Ishikawa Prefectural Nursing University, Kahoku, Japan

[6] Department of Rheumatology, Kanazawa University Hospital, Kanazawa, Japan

[7] Department of Hygiene and Public Health, Graduate School of Advanced Preventive Medical Sciences, Kanazawa University, Kanazawa, Japan

*** Correspondence:**
Shigehiro Karashima, MD, PhD

skarashima@staff.kanazawa-u.ac.jp

Shigefumi Okamoto, DDS, PhD

sokamoto@sahs.med.osaka-u.ac.jp

**Supplementary Material**

**Supplementary Figure 1**. Flowchart on sample selection. For the 234 faecal samples obtained, one sample was obtained per research participant in 2019. As a result of the selection process, a total of 88 samples were available for analysis, of which 46 were female and 42 were male.

**Supplementary Figure 2**. Causal inference between IgG4 levels and bacterial genus by GM. Direct LiNGAM did not reveal networks, arrows, or partial regression coefficients, indicating a causal relationship between GM and IgG4.

**Supplementary Table 1. Immunological characteristics of all participants classified by IgG4.**

| ALL | High IgG4 | Low IgG4 | *P*-value |
|---|---|---|---|
| N | 44 | 44 | |
| Age (years) | 62 ± 11 | 63 ± 10 | 0.858 |
| BMI (kg/m2) | 23.6 ± 3.3 | 23.3 ± 2.7 | 0.690 |
| IgG4 (mg/dl) | 76.6 (59.3-97.3) | 23.8 (17.3-32.3) | <0.001 |
| IgG (mg/dl) | 1406.1 ± 267.2 | 1288.9 ± 259.4 | 0.042 |
| IgE (IU/ml) | 136.5 (47.4-206.0) | 47.1 (24.8-142.5) | 0.041 |
| CH50 (U/ml) | 41.9 (38.5-46.3) | 45.3 (40.2-49.2) | 0.099 |
| Anti-CCP antibody (U/ml) | 0.6 (0.6-0.6) | 0.6 (0.6-0.6) | 1.000 |
| ANA (times) | 40 (40-40) | 40 (40-40) | 1.000 |
| Aniti-SS-A/Ro antibody (U/ml) | 1 (1-1) | 1 (1-1) | 0.894 |
| RF (IU/ml) | 5 (5-8) | 5.5 (5-12) | 0.229 |
| Alcohol consumption (day/week) | 0 (0-4) | 0 (0-3.75) | 0.634 |
| Salt intake (g/day) | 9.7 (8.1-10.6) | 8.9 (7.6-10.5) | 0.307 |
| Smoking (%) | 13.6 | 25.0 | 0.181 |
| Allergy (%) | 11.4 | 11.4 | 1.000 |

The *P*-values were calculated by covariance analysis (ANCOVA or Quade's non-parametric



This is a provisional file, not the final typeset article

ANCOVA). Abbreviations: ANCOVA, analysis by covariance; BMI, body mass index; CCP, cyclic citrullinated peptide; IgG4, Immunoglobulin G4; IgG, Immunoglobulin G; IgE, Immunoglobulin E; CH50, 50% hemolytic unit of complement; ANA, antinuclear antibody; RF, rheumatoid factor.

**Supplementary Table 2. Immunological characteristics of female participants classified by IgG4.**

| female | High | Low | *P*-value |
|---|---|---|---|
| N | 23 | 23 | |
| Age (years) | 61 ± 10 | 63 ± 9.9 | 0.517 |
| BMI (kg/m2) | 23.0 ± 3.3 | 22.6 ± 3.2 | 0.730 |
| IgG4 (mg/dl) | 60.2 (48.0-90.7) | 21.2 (15.9-26.5) | <0.001 |
| IgG (mg/dl) | 1362.1 ± 238.8 | 1347.8 ± 280.6 | 0.857 |
| IgE (IU/ml) | 83.2 (19.2-153.0) | 40.8 (23.5-67.1) | 0.334 |
| CH50 (U/ml) | 42.6 (36.9-47.0) | 47.2 (41.9-51.8) | 0.035 |
| Anti-CCP antibody (U/ml) | 0.6 (0.6-0.6) | 0.6 (0.6-0.6) | 0.977 |
| ANA (times) | 40 (40-40) | 40 (40-40) | 1.000 |
| Aniti-SS-A/Ro antibody (U/ml) | 1 (1-1) | 1 (1-1) | 0.868 |
| RF (IU/ml) | 8 (5-14.5) | 5 (5-7.5) | 0.172 |
| Alcohol consumption (day/week) | 0 (0-0) | 0 (0-1.5) | 0.230 |
| Salt intake (g/day) | 9.1 (8.1-10.7) | 9.2 (8.3-10.2) | 0.754 |
| Smoking (%) | 17.4 | 4.4 | 0.162 |
| Allergy (%) | 13.0 | 8.7 | 0.645 |

The *P*-values were calculated by covariance analysis (ANCOVA or Quade's non-parametric ANCOVA). Abbreviations: ANCOVA, analysis by covariance; BMI, body mass index; CCP, cyclic citrullinated peptide; IgG4, Immunoglobulin G4; IgG, Immunoglobulin G; IgE, Immunoglobulin E; CH50, 50% hemolytic unit of complement; ANA, antinuclear antibody; RF, rheumatoid factor.

**Supplementary Table 3. Immunological characteristics of male participants classified by IgG4.**

| male | High | Low | *P*-value |
|---|---|---|---|
| n | 21 | 21 | |
| Age (years) | 65±11 | 63±11 | 0.544 |
| BMI (kg/m2) | 23.7±2.9 | 24.6±2.3 | 0.300 |
| IgG4 (mg/dl) | 82.1 (75.9-104.0) | 29.8 (23.0-39.6) | <0.001 |
| IgG (mg/dl) | 1419.0±273.1 | 1259.9±262.2 | 0.068 |
| IgE (IU/ml) | 156.0 (81.4-254.0) | 84.2 (45.3-203.0) | 0.365 |
| CH50 (U/ml) | 41.3 (39.1-47.1) | 44.2 (37.5-47.9) | 0.595 |
| Anti-CCP antibody (U/ml) | 0.6 (0.6-0.6) | 0.6 (0.6-0.6) | 0.973 |
| ANA (times) | 40 (40-40) | 40 (40-40) | 0.1545 |
| Aniti-SS-A/Ro antibody (U/ml) | 1 (1-1) | 1 (1-1) | 0.973 |
| RF (IU/ml) | 5 (5-5) | 6 (5-12) | 0.035 |
| Alcohol consumption (day/week) | 3 (0-6) | 3 (0-7) | 0.555 |
| Salt intake (g/day) | 9.6 (8.4-10.5) | 8.5 (7.5-10.8) | 0.465 |
| Smoking (%) | 19.0 | 38.1 | 0.180 |
| Allergy (%) | 4.76 | 19.0 | 0.160 |

The *P*-values were calculated by covariance analysis (ANCOVA or Quade's non-parametric ANCOVA). Abbreviations: ANCOVA, analysis by covariance; BMI, body mass index; CCP, cyclic citrullinated peptide; IgG4, Immunoglobulin G4; IgG, Immunoglobulin G; IgE, Immunoglobulin E; CH50, 50% hemolytic unit of complement; ANA, antinuclear antibody; RF, rheumatoid factor.

**Supplementary Table 4.** All features were selected using the least absolute shrinkage and selection operator logistic models in male.

| Charastaristic | Odds ratio | lower.95ci | upper.95ci | *P*-value |
|---|---|---|---|---|
| male | | | | |

This is a provisional file, not the final typeset article

| | | | | |
|---|---|---|---|---|
| *Romboutsia* | 2.696 | 1.031 | 7.050 | 0.043 |
| *Megasphaera* | 1.678 | 0.984 | 2.861 | 0.057 |
| *[Eubacterium] hallii* group | 0.197 | 0.030 | 1.280 | 0.089 |
| *Veillonella* | 0.205 | 0.032 | 1.311 | 0.094 |
| *Lachnospiraceae* | 0.278 | 0.061 | 1.264 | 0.098 |
| *Fusicatenibacter* | 2.683 | 0.766 | 9.403 | 0.123 |
| *Anaerastipes* | 3.445 | 0.669 | 17.728 | 0.139 |
| *Collinsella* | 0.699 | 0.428 | 1.141 | 0.152 |
| *Parabacteroides* | 1.325 | 0.737 | 2.383 | 0.348 |
| *Bifidobacterium* | 0.930 | 0.734 | 1.177 | 0.546 |
| *Blautia* | 0.973 | 0.783 | 1.209 | 0.805 |
| *[Eubacterium] Coprostanoligenes* group | 0.960 | 0.559 | 1.648 | 0.882 |

**Supplementary Table 5.** Summary of enterobacterial genera: Statistically significant differences were observed in serum IgG4 levels and compositional proportions.

| | all | female | male |
|---|---|---|---|
| ANCOVA | None | None | None |
| TukeyHSD | *Faecalibacterium*<br>*Megasphaera* | *Anaerostipes* | *Faecalibacterium*<br>*Ruminococcus.*1 |
| LEfSe | *Faecalibacterium*<br>*Megasphaera* | *Lachnospiraceae* | *Megasphaera*<br>*[Eubacterium] hallii* group<br>*Faecalibacterium* |
| LASSO | None | None | *Romboutsia* |

| Correlation | *Faecalibacterium* *Megasphaera* *[Eubacterium] hallii* group (Group C) *Lactobacillus* (Group A) | *Megasphaera* *[Eubacterium] hallii* group (Group C) | *Ruminococcus*.1 (Group B) |
|---|---|---|---|

**Supplementary Table 6.** List of Bio sample IDs used for the analysis from the raw sequencing registered in the DNA Data Bank of Japan (DDBJ) (Numbers DRA016467).

| female | | male | |
|---|---|---|---|
| High | Low | High | Low |
| SAMD00621162 | SAMD00621320 | SAMD00621193 | SAMD00621129 |
| SAMD00621329 | SAMD00621335 | SAMD00621234 | SAMD00621217 |
| SAMD00621330 | SAMD00621118 | SAMD00621322 | SAMD00621315 |
| SAMD00621125 | SAMD00621119 | SAMD00621324 | SAMD00621122 |
| SAMD00621128 | SAMD00621138 | SAMD00621328 | SAMD00621126 |
| SAMD00621133 | SAMD00621140 | SAMD00621342 | SAMD00621165 |
| SAMD00621134 | SAMD00621185 | SAMD00621127 | SAMD00621168 |
| SAMD00621137 | SAMD00621190 | SAMD00621153 | SAMD00621169 |
| SAMD00621139 | SAMD00621191 | SAMD00621173 | SAMD00621170 |
| SAMD00621178 | SAMD00621195 | SAMD00621175 | SAMD00621184 |
| SAMD00621188 | SAMD00621201 | SAMD00621180 | SAMD00621192 |
| SAMD00621196 | SAMD00621202 | SAMD00621194 | SAMD00621197 |
| SAMD00621206 | SAMD00621205 | SAMD00621216 | SAMD00621199 |


This is a provisional file, not the final typeset article

| | | | |
|---|---|---|---|
| SAMD00621218 | SAMD00621221 | SAMD00621258 | SAMD00621203 |
| SAMD00621252 | SAMD00621226 | SAMD00621260 | SAMD00621245 |
| SAMD00621265 | SAMD00621228 | SAMD00621261 | SAMD00621257 |
| SAMD00621269 | SAMD00621236 | SAMD00621271 | SAMD00621268 |
| SAMD00621272 | SAMD00621243 | SAMD00621275 | SAMD00621274 |
| SAMD00621284 | SAMD00621259 | SAMD00621282 | SAMD00621278 |
| SAMD00621293 | SAMD00621283 | SAMD00621298 | SAMD00621287 |
| SAMD00621294 | SAMD00621286 | SAMD00621300 | SAMD00621290 |
| SAMD00621301 | SAMD00621288 | | |
| SAMD00621310 | SAMD00621308 | | |
